# Limitation of Unloading in the Developing Grains Is a Possible Cause Responsible for Low Stem Non-structural Carbohydrate Translocation and Poor Grain Yield Formation in Rice through Verification of Recombinant Inbred Lines

**DOI:** 10.3389/fpls.2017.01369

**Published:** 2017-08-08

**Authors:** Guohui Li, Junfeng Pan, Kehui Cui, Musong Yuan, Qiuqian Hu, Wencheng Wang, Pravat K. Mohapatra, Lixiao Nie, Jianliang Huang, Shaobing Peng

**Affiliations:** ^1^National Key Laboratory of Crop Genetic Improvement, MOA Key Laboratory of Crop Ecophysiology and Farming System in the Middle Reaches of the Yangtze River, College of Plant Science and Technology, Huazhong Agricultural University Wuhan, China; ^2^Hubei Collaborative Innovation for Grain Industry Jinzhou, China; ^3^School of Life Sciences, Sambalpur University Sambalpur, India

**Keywords:** grain yield formation, non-structural carbohydrates, source-flow-sink attributes, translocation, rice (*Oryza sativa* L.), unloading of assimilates

## Abstract

Remobilisation of non-structural carbohydrates (NSC) from leaves and stems and unloading into developing grains are essential for yield formation of rice. In present study, three recombinant inbred lines of rice, R91, R156 and R201 have been tested for source-flow-sink related attributes determining the nature of NSC accumulation and translocation at two nitrogen levels in the field. Compared to R91 and R156, R201 had lower grain filling percentage, harvest index, and grain yield. Meanwhile, R201 had significantly lower stem NSC translocation during grain filling stage. Grain filling percentage, harvest index, and grain yield showed the consistent trend with stem NSC translocation among the three lines. In comparison with R91 and R156, R201 had similarity in leaf area index, specific leaf weight, stem NSC concentration at heading, biomass, panicles m^-2^, spikelets per panicle, remobilization capability of assimilation in stems, sink capacity, sink activity, number and cross sectional area of small vascular bundles, greater number and cross sectional area of large vascular bundles, and higher SPAD, suggesting that source, flow, and sink were not the limiting factors for low stem NSC translocation and grain filling percentage of R201. However, R201 had significant higher stem and rachis NSC concentrations at maturity, which implied that unloading in the developing grains might result in low NSC translocation in R201. The results indicate that stem NSC translocation could be beneficial for enhancement of grain yield potential, and poor unloading into caryopsis may be the possible cause of low stem NSC translocation, poor grain filling and yield formation in R201.

## Introduction

Rice is one of the most important food crops for mankind. In recent years, substantial growth of rice consumers, especially in Asia, has increased the demand for overall grain production. The problem has accentuated on account of shrinkage of crop area under cultivation and climate change induced natural calamities. Therefore for food security, emphasis should be given to enhance production through a major revision of crop yield potential (Peng et al., 2008) and yield stability under stress (Deryng et al., 2014).

Since starch represents 80–90% of final grain weight, grain filling and final yield depend on assimilates supplied by current photosynthesis and stem reserve remobilization (Yoshida, 1972). During the vegetative and early reproductive stages, carbon assimilates are temporarily stored in stems and leaf sheaths of cereals as non-structural carbohydrates (NSC) that move to the developing grains at the maturation phase (Scofield et al., 2009). In rice, the apparent contribution of stem NSC to final grain yield has been estimated as 24–27% (Cock and Yoshida, 1972) and 1–28% (Pan et al., 2011). Similar contributions have been recorded for other cereal crops, for example, 11–45% in barley, 13–27% in wheat, and 12–25% in maize (Daynard et al., 1969; Bidinger et al., 1977). In addition, Morita and Nakano (2011) suggested that stem NSC at full heading stage of rice contributes significantly to grain ripening of heat tolerant rice, and preventing grain yield losses. Therefore, enhancing translocation of stem NSC to developing grains is beneficial for grain yield formation. Stem NSC can increase rice yield formation by enhancing degree and rate of grain filling, grain filling percentage, and grain weight (Xue et al., 2008; Yang and Zhang, 2010b). In ratoon rice, NSC reserves in the remaining stem have vital contribution to the speed of vegetative re-growth, flowering, and grain-filling of the second crop (Slewinski, 2012). Recently, Wang et al. (2016) found that stem reserves are most critical for grain yield formation of short-duration rice varieties, suggesting that pre-heading stem NSC should be considered for breeding early maturing rice. Therefore, increasing the pre-anthesis NSC accumulation and the post-anthesis NSC translocation to grains is one of the approaches to improve grain yield of rice.

Sucrose is the main carbohydrate transported over long distances through the phloem from source to sink in most plants (Braun, 2012). Recently, Wang et al. (2015) observed that enhancing sucrose loading can improve rice grain yield by increasing grain size. After heading, stem starch is transformed to sucrose for translocation, enzymes related to starch to sucrose conversion are the regulating factors that affect NSC remobilization in rice (Yang et al., 2001a). The rate-limiting enzymes for the process include α-amylase (EC 3.2.1.1) and β-amylase (EC 3.2.1.2) (Ishimaru et al., 2004) for starch degradation, and SPS (EC 2.4.1.14) and sucrose synthase (EC 2.4.1.13) for sucrose synthesis (Persia et al., 2008; Verma et al., 2011). Therefore, high activities of the four enzymes are beneficial for NSC translocation and sequential improvement of grain yield.

The transport pathway and sink characters have been observed to influence stem NSC re-partition. It may be possible that number and area of vascular bundles determine the efficiency of NSC translocation and grain yield formation in a genotype (Cui et al., 2003; Liu et al., 2008; Terao et al., 2010; Pan et al., 2011). Several enzymes, such as invertase (EC 3.2.1.26), sucrose synthase (EC 2.4.1.13), and ADP-glucose pyrophosphorylase (AGPase, EC 2.7.7.27), are considered as key enzymes linked to sucrose to starch conversion in developing grains (Yang et al., 2004; Braun et al., 2014). Rapid sucrose to starch conversion may enhance carbohydrate translocation into grains; however, the effects of these enzymes on stem NSC translocation into grains are not well understood.

Because grain yield capacity sustains on carbohydrate partitioning, assimilate remobilization from stems and leaf sheaths becomes crucial in exigency, when challenging environments slacken current photosynthesis. NSC re-translocation associates with stress tolerance such as high or low irradiance (Okawa et al., 2003), water deficiency stress (Yang et al., 2001a; Gupta et al., 2011), heat stress (Tahir and Nakata, 2005; Morita and Nakano, 2011; Shi et al., 2013; Xiong et al., 2015) and low nitrogen availability (Pan et al., 2011). These results illustrate the importance of stem NSC for yield stability and stress resistance in cereal crops.

Attempts have also been made to explore genomic expression responsible for the mechanism of NSC partitioning. Currently several yield enhancing QTLs have been validated in rice (Xing and Zhang, 2010). Although grain yield related traits sustain on the quantum of assimilate partitioning, knowledge is scant on the genomics determining the nature of transport. Takai et al. (2005) identified presence of a QTL for increased grain filling percentage on chromosome 8, which exactly overlapped with that for NSC content in culms and sheaths during grain filling, and the allele from Milyang 23 for increasing grain filling percentage was linked with decreased stem NSC content. Similarly, the QTL *tgw6* on chromosome 6 improves pre-heading carbohydrates storage capacity in the leaf sheaths of rice that ultimately increases yield potential (Ishimaru, 2003). For most of the identified QTLs, there is lack of information on the functions of various components of source-sink-flow system and the mechanism for controlling NSC accumulation and translocation in terms of phenotype.

In our previous study, we located two QTLs associated with stem NSC transferred to grains using recombinant inbred lines, and identified three lines with or without the two QTLs, which show contrasting characters of stem NSC transport to grains and grain filling percentage (Pan, 2010). In this study, attempts were made to clarify the underlying physiological causes for the differences in contribution of stem NSC translocation to grain filling among the three lines.

## Materials and Methods

### Plant Materials and Field Experiments

Rice (*Oryza sativa* L.) recombinant inbred lines from a cross between Zhenshan 97 and Minghui 63 were used for QTL location related to yield and NSC translocation in the previous experiments (Pan, 2010). Three lines (R91, R156 and R201) were selected by screening for QTLs for possible control on NSC accumulation and translocation, such as, the marker intervals G359-R753-C161 and RG360-R3166 in chromosome 1 and chromosome 5, respectively, at which the alleles are from Zhenshan 97 (Pan, 2010). The two intervals were associated with grain yield traits such as grains per panicle, yield per plant, and grain weight (Xing et al., 2002; Xing and Zhang, 2010). R91 carries the interval RG360-R3166, R156 contains the interval G359-R753-C161, and R201 has none of the two intervals. The three lines have the similar growth duration (approximately 82 days from seeding to heading date in Wuhan) and plant heights of 101.1, 96.8, and 99.3 cm, respectively (Pan, 2010).

Field experiments were conducted in farmers’ fields at Dajin County (29°51′N, 115°33′E), Wuxue city, Hubei province, China, during the rice growing season from May to October in 2013 and 2014. Average solar radiation was 11.5 MJ m^-2^ d^-1^, and average maximum and minimum temperatures were 23.4 and 15.2°C during the rice-growing season, respectively. The experimental fields had a gleyed type soil and soil samples taken from the upper 20 cm had the following properties: pH 5.3, 26.3 g kg^-1^ organic matter, 2.0 g kg^-1^ total N, 8.8 mg kg^-1^ Olsen P, and 114.1 mg kg^-1^ exchangeable K.

The plants of each line were grown in individual plot size of 12 m^2^. The plots were arranged in a split plot design with three replications. Because nitrogen dynamics is linked strongly to carbon assimilation and translocation in rice (Takai et al., 2005), R91, R156 and R201 were grown at two levels of nitrogen: LN without N fertilizer application and HN with N fertilization. Fertilizers were applied at the rates of 150 kg N ha^-1^ as urea, 40 kg P ha^-1^ as calcium superphosphate, and 100 kg K ha^-1^ as potassium chloride, respectively. Nitrogen fertilizer was split-applied: 60 kg ha^-1^ at basal (1 day before transplanting), 30 kg ha^-1^ at early tillering stage (7 days after transplanting), and 60 kg ha^-1^ at panicle initiation. Phosphate fertilizer was applied at basal, and K fertilizer was split equally as basal (50 kg K ha^-1^) and top dressing at the panicle initiation (50 kg K ha^-1^).

Pre-germinated seeds were sown in a seedbed during the second week of May in 2013 and 2014. Seedlings were transplanted to the experimental plot after 4 weeks, at a hill spacing of 0.13 m × 0.30 m. The main plots were separated with double bunds to prevent water flow between the N applications, and all the bunds were covered with plastic film, extending to a depth of 20 cm below the soil surface. Pests, diseases, birds, and weeds were intensively controlled to avoid yield loss.

### Determination of Leaf Area, SPAD Value and Vascular Bundle Traits

At heading stage, six plants were sampled from each plot, and then were separated into leaf blades, stems (sheaths and culms) and young panicles. Leaf area was measured by LI-3100 Area Meter (LI-COR, United States). All parts were oven-dried at 80°C to constant dry weight. Leaf area index (LAI, m^2^ m^-2^) and specific leaf weight (SLW, g m^-2^) were calculated.

For evaluation of leaf green-stay and chlorophyll content, SPAD values were determined at the midpoint of the full leaf from top (1.5 leaf) at panicle initiation, flag leaf at heading date and every 10 days thereafter after heading until maturity using SPAD-502 chlorophyll meter (Konica Minolta Holdings, Inc., Japan) from five hills.

Six panicles of main stems, which emerged on the same day, were collected from each plot. Peduncle of 1 cm length below the neck node of the panicle was excised and fixed in formalin-acetic acid-alcohol for measuring number and cross sectional area of vascular bundles. The transverse hand section about 1-mm thick was made with a razor blade, and mounted on a slide. The sections were photographed under an inverted microscope (Nikon Corporation, Inc., Japan) to record the numbers of large and small vascular bundles (LVB and SVB). The pictures were used for measurement of the cross sectional area of the vascular bundle using Photoshop CS5 Software package (Adobe Systems, Inc., United States).

### Determination of Yield and Its Components

Six plants in the middle of each plot were harvested for measuring yield traits at maturity. Plants were separated into leaf blades, stems (sheaths and culms) and panicles. After recording panicle number, all spikelets of the panicles were hand-threshed, and then separated into filled and unfilled grains (including half filled and empty grains) by submerging them in tap water. The unfilled grains floating at the surface of water were separated into partially filled and empty grains by both pressing each of them between the thumb and the forefinger and checking after opening lemma and palea. The empty grains were considered as sterile ones. After oven-dried, all filled grains were then weighted, and three sub-samples of 30 g filled grains were used to determine the grain numbers. Similarly, all of the partially filled and empty grains were also counted, respectively. And then, 1000-grain weight (g), percentage of filled and half filled grains (%) were calculated.

All the leaves, stems (culms and sheaths), panicle rachis, filled grains, half-filled and empty grains were separately collected and oven-dried at 80°C to constant weight. Total biomass (t ha^-1^) was the total dry weight of the aboveground parts. Panicles per m^2^, spikelets per panicle, and grain yield (t ha^-1^) were calculated. Sink capacity (g m^-2^) was calculated as spikelets per m^2^ multiplied by average grain weight. The harvest index (HI, %) was calculated as the percentage of dry matter of filled grain to total aboveground biomass.

### Determination of Soluble Sugar and Starch Contents

Contents of soluble sugars and starch were determined according to Yoshida et al. (1976). Oven-dried plant samples were ground to powder and filtered through a 1-mm sieve. Approximately 100 mg of the sample was extracted with 80% aqueous ethanol at 80°C for 30 min. The extract was centrifuged and the supernatant was transferred to a 100 mL volumetric flask. The extraction process was repeated three times; all the three supernatants were pooled in the flask, following by addition of distilled water to 100 mL. Aliquot of the extract was used for determination of soluble sugars with anthrone reagent.

For the starch determination, the residue after centrifugation in the tube was added 2 mL of distilled water, and put in a boiling water bath for 15 min. Two milliliter of 9.2 mol L^-1^ HClO_4_ was added to the tube and put into ice bath for 15 min for complete digestion of starch into glucose. Supernatant of the extract was collected in a 100 mL volumetric flask after centrifugation. The extraction was repeated by putting the residue in 2 mL of 4.6 mol L^-1^ HClO_4_ for 15 min for a second time. The supernatants were pooled together in the flask, and then added distilled water to 100 mL. For the colorimetric assay optical density was measured at 620 nm on a microplate reader (Nano Quant, infinite M200, Tecan, Switzerland). Glucose released in the extraction was estimated with anthrone reagent and converted to starch value by multiplying by 0.9 (Pucher et al., 1932).

The concentrations of soluble sugars and starch expressed as mg glucose g^-1^ dry weight were calculated by comparing with glucose standard. The NSC concentration of a given plant part refers to sum of the concentrations of soluble sugars and starch (mg glucose g^-1^ dry weight). The total mass of NSC stored in stems (TM, g m^-2^) was calculated as stem biomass multiplied by the NSC concentration. The ratio of stem NSC to rachis NSC was calculated on the basis of NSC concentration of the organs. The amount of stem NSC per spikelet was defined as the ratio of total of stem NSC accumulation per m^2^ to spikelets per m^2^. The calculations related to stem NSC translocation are as follows: apparent transferred mass of NSC from stems to grains (ATM, g m^-2^) is the difference of TM at heading and TM at maturity; apparent ratio of transferred NSC from stems to grains (AR, %) is the ratio of ATM to TM at heading; apparent contribution of transferred NSC to grain yield (AC, %) is the ratio of ATM to grain yield.

### Extraction and Assays of Enzymes for Starch to Sucrose Conversion in Stems

The four main stems from four plants were sampled at 7 days after heading, and were frozen by liquid nitrogen and stored at -80°C for enzyme assays. The activities of α-amylase (EC 3.2.1.1) and β-amylase (EC 3.2.1.2) were determined according to the methods of Bhatia and Singh (2002). Briefly, the stems were ground using 100 mM phosphate buffer (pH 6.5) and centrifuged at 4°C and 12,000 rpm for 20 min, the supernatant was used for the assay of the two amylases.

For α-amylase, 0.1 mL of enzyme preparation was added to 0.6 mL of 12.7 mM calcium acetate (pH 6.0) and 0.3 mL of 0.067% soluble starch solution. The assay mixture was incubated at 70°C for 20 min to inactivate β-amylase and then incubated at 37°C for 20 min; the intensity of color developed with 0.1% I_2_–1% KI was measured at 610 nm. The control was conducted with inactive enzyme by heating in boiling water for 30 s. The amount of remained starch in the mixture was calculated by comparison to a standard, respectively.

For β-amylase, 200 mM sodium acetate (pH 3.6) containing 0.1 mM ethylenediaminetetraacetic acid (EDTA) was used instead of 12.7 mM calcium acetate (pH 6.0) for inhibiting α-amylase activity. Other procedures were the same as described above. Protein content in the enzyme preparation was measured according to Bradford (1976) using bovine serum albumin as the standard. The amount of hydrolyzed starch was the difference in starch amount between the control and assay mixture. The activities of α-amylase and β-amylase were calculated as mg starch hydrolyzed mg^-1^ protein h^-1^.

For sucrose phosphate synthase (SPS, EC 2.4.1.14) and sucrose synthase in the synthetic direction (SSs, EC 2.4.1.13) measurements, the stems were ground in a medium containing 50 mM Hepes-NaOH (pH 7.5), 10 mM MgCl_2_, 1 mM EDTA, 5 mM dithiothreitol (DTT), 1 mM phenazine methosulfate (PMSF), 1 mM benzamidine, and 3% (w/v) polyvinylpolypyrrolidone (PVPP). The homogenate was then centrifuged at 4°C and 12,000 rpm for 20 min, the supernatant was used for the assay of the two enzymes. The activity of SPS was determined as described previously by Rufty et al. (1983) with modification. Thirty microliter of enzyme preparation was added to 70 μL of medium containing 50 mM Hepes-NaOH (pH 7.5), 3.5 mM UDP-glucose, 3.5 mM fructose-6-P, and 10 mM MgCl_2_. After incubating at 30°C for 15 min, the reactions were terminated by addition of 100 μL of 1.0 M NaOH. The unreacted fructose-6-P was destroyed by placing the reaction mixture in boiling water for 10 min. After cooling, 0.75 mL of 9 mol L^-1^ HCl and 0.25 mL of 0.1% (w/v) resorcinol were added, and the mixture was incubated at 80°C for 10 min, following by the absorbance measurement at 480 nm. The control was conducted without UDP-glucose. The amount of sucrose synthesized was calculated via comparison to the standard.

For SSs activity, 30 μL of enzyme preparation and 30 μL of 100 mM fructose were added into 70 μL of medium containing 100 mM Hepes-NaOH (pH 8.5), 5 mM KCl, 5 mM NaCl, and 8 mM UDP-glucose, and then the reaction mixture was incubated at 30°C for 15 min (Wardlaw and Willenbrink, 1994), the following procedures were the same as described above. The SPS and SSs activities were expressed as μmol sucrose synthesized mg^-1^ protein h^-1^, respectively.

### Extraction and Assays of Enzymes for Sucrose to Starch Conversion in Grains

The grains attached on the top primary branches of panicle were sampled at 7 days after heading from plants grown in the experimental fields in 2014, and frozen directly in liquid nitrogen and stored at -80°C. The extraction and assay for AGPase (EC 2.7.7.27) was carried out according to Nakamura et al. (1989). Briefly, the frozen grains were homogenized with extraction medium, which contained 100 mM Hepes-NaOH (pH 7.6), 8 mM MgCl_2_, 5 mM DTT, 2 mM EDTA, 12.5% (v/v) glycerol, and 5% (w/v) PVPP. The homogenate was centrifuged at 4°C and 12,000 rpm for 20 min, and the supernatant was considered as enzyme extraction for the enzyme activity determination. Twenty microliter of enzyme extraction was added to 110 μL of medium in an Eppendorf tube, which contained 100 mM Hepes-NaOH (pH 7.4), 1.2 mM ADP-glucose, 3 mM PPi, 5 mM MgCl_2_, 4 mM DTT, and then incubated for 15 min at 30°C. The reaction was terminated by putting the tube into boiling water for 30 s. The resulting solution was transferred to another tube and centrifuged at 12,000 rpm for 10 min. The supernatant of 100 μL was taken and mixed with 3 μL of 10 mM nicotinamide adenine dinucleotide phosphate (NADP). After addition of phosphoglucomutase (0.4 unit) and glucose dehydrogenase (0.35 unit), the absorbance was measured at 340 nm. The control was conducted without substrates. After comparison to a standard curve, activities of AGPase were expressed as μmol NADPH mg^-1^ protein h^-1^.

For sucrose synthase activity in the cleavage direction (SSc, EC 2.4.1.13), acid and neutral invertase (AI and NI, EC 3.2.1.26), the frozen grains were ground in a extracting buffer composed of 150 mM Tris–HCl (pH 8.0), 2 mM EDTA, 10 mM MgCl_2_, 0.1 mM PMSF, 1 mM benzamidine, 10 mM ascorbic acid, and 3% (w/v) PVPP. The homogenate was centrifuged at 12,000 rpm for 20 min and the supernatant was used for the enzyme assays. All the extracting procedures were carried out at 4°C.

The AI was assayed in 0.3 mL of 100 mM sodium acetate buffer (pH 4.8), 0.1 mL of 100 mM sucrose, and 0.1 mL of enzyme preparation as described previously (Pan et al., 2005). The reaction mixtures were incubated for 15 min at 30°C and were terminated by boiling for 1 min. Then 0.5 mL of 3,5-dinitrosalicylic acid was added, following by incubating for 5 min at 100°C. The absorbance was measured at 540 nm after cooling. The control was conducted by adding 0.1 mL distilled water instead of substrate (sucrose). For NI, 40 mM phosphate buffer (pH 7.0) was used instead of 100 mM sodium acetate buffer (pH 4.8), other procedures were the same as described above.

The SSc was assayed according to (Ranwala and Miller, 1998) with modification. Reaction medium contained 0.1 mL of enzyme extraction, 0.1 mL of 100 mM sucrose, and 0.3 mL of reaction buffer that consisted of 100 mM Hepes-NaOH (pH 5.5), 5 mM NaCl, and 1.8 mM UDP. Reaction proceeded at 30°C for 15 min and was terminated by boiling for 1 min. Then 0.5 mL of 3,5-dinitrosalicylic acid was added, following by incubating for 5 min at 100°C. The absorbance was measured at 540 nm after cooling. The control was conducted without substrates. The activities of SSc, AI and NI were expressed as μmol glucose produced mg^-1^ protein h^-1^.

We compared enzyme activities (SPS, SSs, AI, NI, SSc and AGPase) measured using different controls (the control with H_2_O instead of substrate and the control with corresponding substrate plus inactive enzyme extraction) in R156 under low nitrogen condition at 7 days after heading in another field experiment in 2016, respectively (Supplementary Tables S1, S2). The data shows that the three substrates (UDP-glucose, sucrose, and ADP-glucose) used in enzyme activity assays were free of the expected products (Supplementary Table S1). Additionally, the data indicate that enzyme activities were not significantly different between two different controls (Supplementary Table S2). Therefore, using enzyme extraction with H_2_O instead of substrate as control was appropriate, and did not result in overestimation or underestimation for enzyme activities.

### Statistical Analysis

Means over the 2 years were used for statistic analyses, considering the similar performances of the investigated traits in 2013 and 2014. For LAI, SLW, and SPAD that were only measured in 2014, means over the three replications were used. Data are shown as mean ± standard error. One-way analysis of variance was carried out, then the least significant difference test (LSD test) was used for estimation of the difference significance for mean of investigated traits between the two N levels for the same line, and among the three lines under an identical N level at the 5% level, using Statistix 9 software package (Analytical software, Tallahassee, FL, United States).

## Results

### Yield and Its Components

Compared to R91 and R156, R201 had similar biomass, panicles m^-2^, spikelets per panicle and sink capacity under LN and HN, but grain yield and harvest index of R201 was lower on account of poor grain filling under LN and HN (**Table 1**), and nearly half of the grains in R201 remained un-filled or poorly filled. HN improved grain filling percentage of R201 resulting in increased HI and grain yield, although it could not reach the level of the other two lines. In sharp contrast to R201, the other two lines did not respond to HN in yield parameters. There was no corresponding effect of grain yield and HI values in R91 and R156.

**Table 1:** Yield and yield components of rice R91, R156 and R201 under low (LN) and high nitrogen (HN) conditions.

Treatment	Line	Biomass (t ha^-1^)	SC (g m^-2^)	Panicle (No. m^-2^)	Spikelet (No. panicle^-1^)	KGW (g)	HF (%)	GFP (%)	HI (%)	Yield (t ha^-1^)
LN	R91	12.3 ± 0.5a	724 ± 19a	247 ± 8a	130 ± 1aˆ*	22.9 ± 0.2cˆ*	15.6 ± 0.7b	75.2 ± 1.3b	44.3 ± 0.5a	5.4 ± 0.2a
	R156	12.1 ± 0.3a	672 ± 20ab	241 ± 5a	108 ± 2b	25.9 ± 0.1a	8.1 ± 0.3c	83.1 ± 0.5aˆ*	46.1 ± 0.3a	5.6 ± 0.1a
	R201	11.6 ± 0.4a	641 ± 7b	235 ± 6a	116 ± 4bˆ*	23.9 ± 0.1b	48.3 ± 1.3aˆ*	42.4 ± 1.4c	23.6 ± 0.5b	2.8 ± 0.1b
HN	R91	13.3 ± 0.4aˆ*	811 ± 21a	297 ± 10aˆ*	122 ± 1a	22.4 ± 0.1c	19.9 ± 1.5b	69.9 ± 2.7a	42.8 ± 0.8a	5.7 ± 0.2a
	R156	12.2 ± 0.3b	718 ± 21b	275 ± 11a	102 ± 4b	25.7 ± 0.3a	14.9 ± 0.3bˆ*	75.3 ± 0.8a	44.3 ± 0.8a	5.4 ± 0.2a
	R201	12.4 ± 0.2b	728 ± 23b	276 ± 15a	110 ± 3ab	24.1 ± 0.1b	32.8 ± 1.5a	56.7 ± 0.7bˆ*	33.4 ± 1.2bˆ*	4.1 ± 0.1b^∗^

*SC, Sink capacity; KGW, 1000-Grain weight; HF, Half filled grain percentage; GFP, Grain filling percentage; HI, Harvest index. Different letters following mean ± standard error indicate significant difference among the three lines under an identical N level at *p* < 0.05. ^∗^Indicates significant difference for the same line between the two N conditions at *p* < 0.05.*

### NSC Concentrations in Stems and Rachises

**FIGURE 1:**
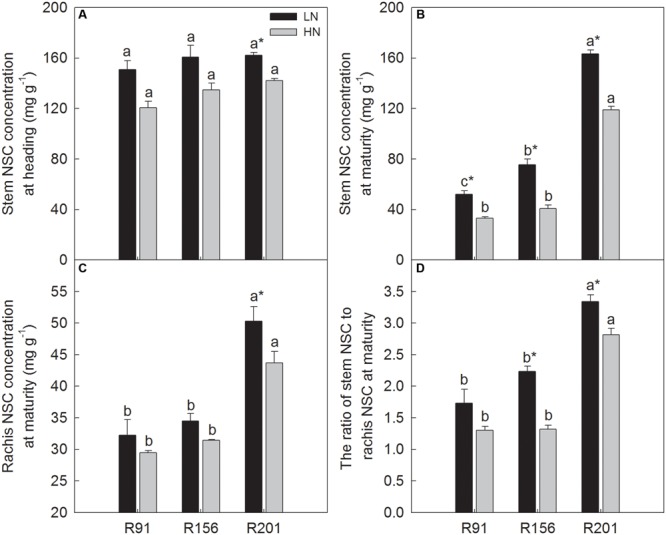
The non-structural carbohydrates (NSC) concentration in stems **(A,B)** and rachis **(C)** and the ratio of stem NSC to rachis NSC **(D)** of rice R91, R156 and R201 under low (LN) and high nitrogen (HN) conditions at heading and maturity. Different letters placed on top of histograms denote significant difference at *p* < 0.05 under the same nitrogen condition. ^∗^Indicates significant difference for the same line between LN and HN at *p* < 0.05. Data are shown as mean ± standard error.

At heading, stem NSC concentration did not differ significantly among the three lines used under LN and HN, respectively (**Figure 1A**). HN depressed the concentration identically in all of them. However, stem NSC concentration of R201 at maturity was about two times higher than the other two lines under the two N applications, respectively (**Figure 1B**). Similar to the response at heading, HN treatment depressed stem NSC significantly at maturity, but the margin of depression was less in R201, compared to the other two lines (27.1% in R201, 36.6% in R91 and 45.9% in R156). In R91 and R156, stem NSC concentration at maturity was significantly lower compared to that at heading, but the progression of time had no effect on the concentration in R201.

The effects of nitrogen application on rachis NSC concentration were similar in the three lines (**Figure 1C**), however, R201 had higher rachis NSC concentration than that in R91 and R156. Additionally, it is noticeable that the NSC concentration ratios of stem to rachis were 3.3 and 2.8 in R201 under LN and HN, respectively, which were significantly higher than those in R91 (1.7 and 1.3) and R156 (2.2 and 1.3) (**Figure 1D**). NSC concentrations ratios of stem to rachis were higher under LN than those under HN in the three lines.

### NSC Translocation

**FIGURE 2:**
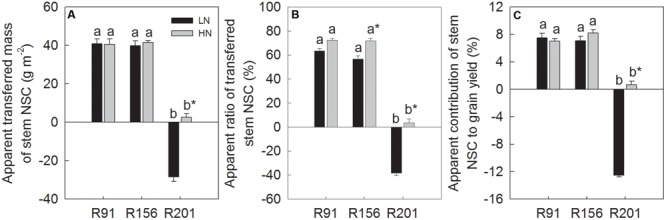
The apparent transferred mass **(A)** and apparent ratio **(B)** of stem NSC, and apparent contribution of stem NSC to grain yield **(C)** of rice R91, R156 and R201 under low (LN) and high nitrogen (HN) conditions during grain filling period. Different letters placed on top of histograms denote significant difference at *p* < 0.05 under the same nitrogen condition. ^∗^Indicates significant difference for the same line between LN and HN at *p* < 0.05. Data are shown as mean ± standard error.

During the grain filling period, the translocation traits represented by ATM, AR and AC showed high NSC partitioning from the stems into the grains in R91 and R156 that possessed good grain filling traits under LN. But these values were negative in line R201 possessing no such trait advantage for translocation under LN, implying no net translocation from stems to grains and more accumulation of stem NSC. Generally, nitrogen application did not increase NSC partitioning in R91 and R156. In contrast, nitrogen application enhanced stem NSC translocation, compared to that under LN in R201, but the level was not as high as that of the other two lines (**Figures 2A–C**).

### Leaf Characteristics for Source Activity

**FIGURE 3:**
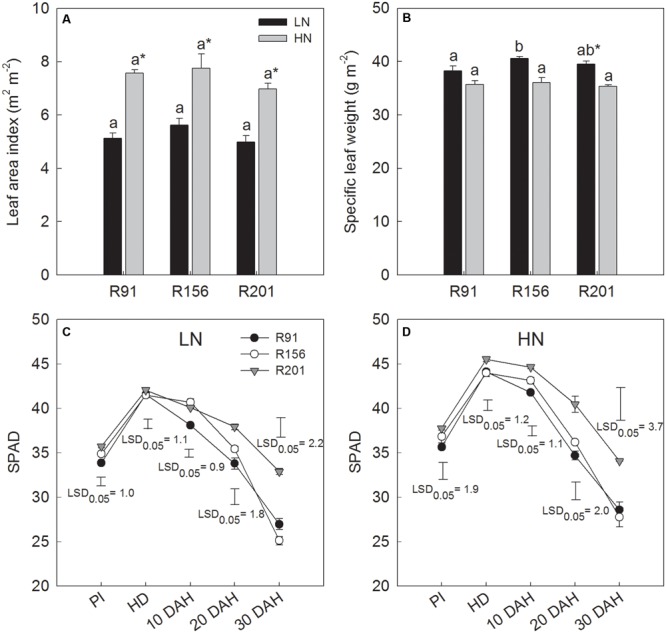
The leaf area index **(A)**, specific leaf weight **(B)** at heading stage and dynamic of SPAD values **(C,D)** during grain filling of rice R91, R156 and R201 under low (LN) and high nitrogen (HN) conditions. Different letters placed on top of histograms denote significant difference at *p* < 0.05 under the same nitrogen condition. ^∗^Indicates significant difference for the same line between LN and HN at *p* < 0.05. Vertical bars indicate LSD_0.05_ for comparing the means among the three lines at an identical stage or date. PI, panicle initiation; HD, heading date; DAH, day after heading. Data are shown as mean ± standard error.

There was no difference in LAI values at heading of the three lines under LN and HN, respectively (**Figure 3A**). The difference in SLW at heading between them was minimal under LN and HN, respectively (**Figure 3B**). Nitrogen application significantly increased LAI in all three lines with no difference in quantum of response. Conversely, nitrogen application depressed SLW marginally in all the three lines and the effect was significant only in R201, compared to that under LN.

SPAD value increased from panicle initiation to heading and declined progressively with time till 30 days after heading in all three lines under both LN (**Figure 3C**) and HN (**Figure 3D**). The value increased with application of nitrogen (HN) in all lines. Among the lines, SPAD value was higher in R201, compared to the other two lines under LN and HN, especially during the late stage of grain filling, implying that R201 stayed green longer.

### Activities of Enzymes for Starch to Sucrose Conversion in Stems

**FIGURE 4:**
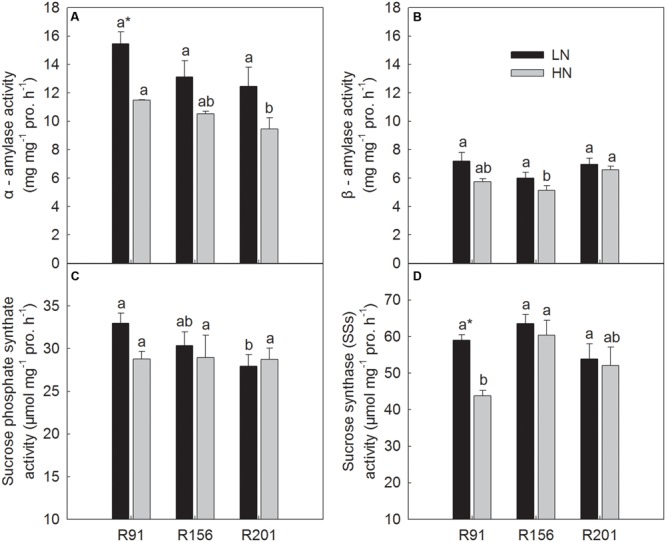
Activities of α-amylase **(A)**, β-amylase **(B)**, sucrose phosphate synthase **(C)**, and sucrose synthase in the synthetic direction **(D)** in stems of rice R91, R156 and R201 under low (LN) and high nitrogen (HN) conditions. Different letters placed on top of histograms denote significant difference among the three lines at *p* < 0.05 under the same nitrogen condition. ^∗^Indicates significant difference for the same line between LN and HN at *p* < 0.05. Data are shown as mean ± standard error.

The activities of α-amylase, β-amylase, SPS, and SSs were presented in **Figure 4**. Under LN, the activities of α-amylase, β-amylase, and SSs showed no substantial differences among R91, R156 and R201, respectively (**Figures 4A,B,D**). The activity of SPS in R201 was similar as that in R156, which was lower than that in R91 (**Figure 4C**).

Under HN, R201 had the similar α-amylase activity as R156, which was lower than that in R91 (**Figure 4A**), and similar β-amylase activity was observed in R201 and R91, which was higher than that of R156 (**Figure 4B**). There was no difference in SPS activity among the three lines under HN (**Figure 4C**). R201 had the similar SSs activity as R156, which was slightly higher than that in R91 (**Figure 4D**).

In general, nitrogen treatment had no effects on activities of the three enzymes (α-amylase, β-amylase, SPS) in the three lines, respectively; only significant difference was observed in activity of α-amylase and SSs in R91 between LN and HN (**Figures 4A,D**).

### Vascular Bundles

**FIGURE 5:**
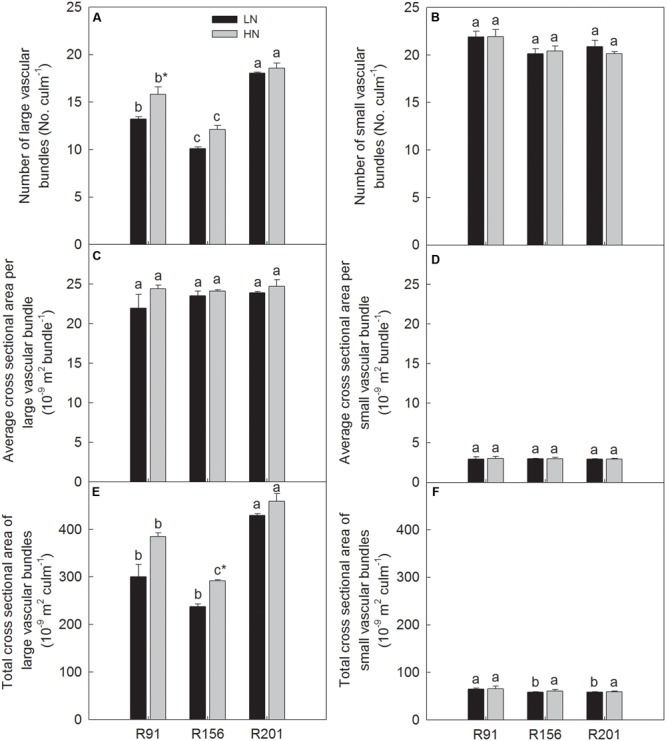
Number **(A,B)**, average cross sectional area **(C,D)**, total cross sectional area **(E,F)** of large and small vascular bundle of rice R91, R156 and R201 under low (LN) and high nitrogen (HN) conditions. Different letters placed on top of histograms denote significant difference at *p* < 0.05 under the same nitrogen condition. ^∗^Indicates significant difference for the same line between LN and HN at *p* < 0.05. Data are shown as mean ± standard error.

Among the lines used, R201 had the highest number and total cross sectional area of LVB, and both the parameters declined significantly in R91 followed by R156 under LN and HN (**Figures 5A,E**), respectively. Average cross sectional area of LVB did not differ significantly among the three lines used (**Figure 5C**). There was no substantial difference in number of SVB (**Figure 5B**) and average cross sectional area of SVB (**Figure 5D**) among the three lines under LN and HN, respectively. R201 and R156 had the same total cross sectional area of SVB, and lower than that in R91 under LN, however, the three lines had the same total cross sectional area of SVB under HN (**Figure 5F**). Generally, nitrogen application did not substantially change the six traits related to vascular bundle but number of LVB in R91 (**Figure 5A**) and total cross sectional area of LVB in R156 (**Figure 5E**).

**FIGURE 6:**
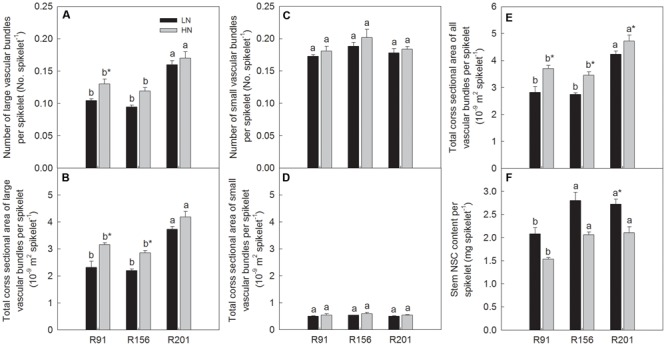
The ratio of number **(A,C)** and cross sectional area of vascular bundle **(B,D,E)**, and non-structural carbohydrates (NSC) content to spikelet **(F)** of rice R91, R156 and R201 under low (LN) and high nitrogen (HN) conditions. Different letters placed on top of histograms denote significant difference at *p* < 0.05 under the same nitrogen condition. ^∗^Indicates significant difference for the same line between LN and HN at *p* < 0.05. Data are shown as mean ± standard error.

Number of LVB per spikelet and total cross sectional area of LVB per spikelet was highest in R201 and it was followed by R91 and R156 sequentially under two nitrogen applications, respectively (**Figures 6A,B**), and no differences could be noticed in SVB under both nitrogen applications (**Figures 6C,D**). Total cross sectional area of LVB and SVB per spikelet was highest in R201 and it declined sequentially in R91 and R156 under LN and HN, respectively (**Figure 6E**). Stem NSC content per spikelet was high in both R201 and R156 lines, but low in R91 (**Figure 6F**).

Nitrogen application significantly increased total cross sectional area of LVB and SVB per spikelet (**Figure 6E**). No changes could be observed in number of SVB per spikelet and total cross sectional area of SVB per spikelet between LN and HN (**Figures 6C,D**), N application increased number of LVB per spikelet and total cross sectional area of LVB per spikelet, but the significant difference did not observed in all lines used (**Figures 6A,B**). Nitrogen treatment depressed stem NSC content per spikelet in all lines (**Figure 6F**).

### Activities of Enzymes for Sucrose to Starch Conversion in the Developing Grains

**FIGURE 7:**
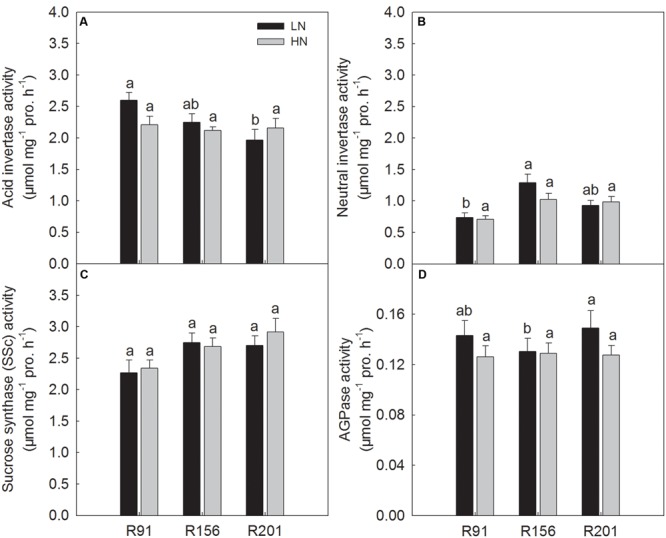
Activities of acid invertase **(A)**, neutral invertase **(B)**, sucrose synthase in the cleavage direction **(C)**, and AGPase **(D)** in developing grains of rice R91, R156 and R201 under low (LN) and high nitrogen (HN) conditions. Different letters placed on top of histograms denote significant difference among the three lines at *p* < 0.05 under the same nitrogen condition. Data are shown as mean ± standard error.

In general, the activities of the four enzymes involved in sucrose-to-starch conversion in filling grains, SSc, AI, NI, and AGPase, were not significantly different among the three lines under HN, respectively (**Figures 7A–D**). Under LN, R201 had the similar AI activity as R156, which was lower than that of R91 (**Figure 7A**). Similar NI activity was also observed in R201 and R156 under LN, which was slightly higher than that of R91 (**Figure 7B**). There was no substantial difference in SSc activity among the three lines under LN (**Figure 7C**). R201 had the similar AGPase activity as R91, which was higher than that of R156 under LN (**Figure 7D**). Generally, nitrogen treatment had no substantial effects on activities of the four enzymes in an identical line.

## Discussion

### Contribution of Stem NSC Translocation to Yield Formation

Yang and Zhang (2010a) proposed that post anthesis management of crop growth was essential for enhancement of grain yield in rice, in which remobilization of pre-stored assimilate reserves from vegetative tissues to grains could be a limiting factor for grain filling, especially under challenging environments. Also remobilization of stem and leaf sheath NSC becomes critical when grain yield potential is sustained on high biomass accumulation as in the large-panicle super rice (Fu et al., 2011). In line with these observations, our study reiterates the vital contribution of stem NSC to yield formation of rice. It revealed tightly positive association between NSC translocation with grain filling percentage, harvest index and grain yield in the three lines tested (**Figures 2A–C** and **Table 1**). Similarly to wheat (Shearman et al., 2005) and rice (Takai et al., 2005; Pan et al., 2011), enhancement of post anthesis NSC translocation to grains could be one of the approaches for better yield performance of rice. However, we did not observe significant correlation of total transferred NSC with stem NSC concentration (*r* = -0.22 under LN and -0.56 under HN, *n* = 9, *p* > 0.05), total stem NSC accumulation (*r* = -0.28 under LN and 0.4 under HN, *n* = 9, *p* > 0.05), and total biomass (*r* = 0.35 under LN and 0.43 under HN, *p* > 0.05) at heading. These results suggest that high NSC accumulation do not always imply more translocation of NSC during grain filling, and other factors may affect re-distribution of stem NSC.

On the other hand, we observed that amount of stem NSC per spikelet had significant and positive correlation with grain weight (*r* = 0.68 under LN and 0.78 under HN, *p* < 0.05). Fu et al. (2011) reported that stem NSC per spikelet at heading were significantly and positively correlated with grain filling rate and grain weight of inferior spikelets, and NSC per spikelet at heading significantly increased when nitrogen was applied at the spikelet differentiation stage. Other management practices like controlled soil drying, alternate wetting and moderate soil drying are the possible methods that may encourage greater partitioning of assimilates and be beneficial for grain yield formation (Yang and Zhang, 2010a). Therefore, the stem NSC per spikelet parameter deserves more attention in the future.

### Possible Reasons for Poor Yield Performance in R201

Compared to R91 and R156, R201 had poor yield performance (**Table 1**). Yamamoto et al. (1991) reported that spikelet number per panicle correlated significantly and negatively with grain filling percentage among 13 rice varieties examined. In the present study, R201 had more spikelets per panicle than R156 by 7%, and less by about 10% in comparison with R91 (**Table 1**), suggesting that spikelets per panicle may not be the major cause for poor grain filling percentage in R201. On the other hand, low grain filling percentage mainly results from increase of sterile spikelets (Rao et al., 2008). In our study, R201 shows a similar sterile spikelet percentage (approximately 10%), as observed for R91 and R156 (**Table 1**), but it shows a higher half-filled grain percentage. These results suggest that poor filling of the fertilized spikelets on account of meager assimilate translocation in the post-anthesis period contributed to lower grain filling percentage and grain yield in R201.

Because more than 70% of grain yield comes from assimilates produced in the post-anthesis period (Cock and Yoshida, 1972; Pan et al., 2011), a high photosynthetic rate during grain filling is desirable to sustain source activity. In addition to photosynthetic rate, several parameters, such as LAI, SLW, and SPAD value, are often used to describe the photosynthetic capacity. Leaf thickness is associated with SLW, and a thick leaf usually has high chlorophyll and nitrogen content, and amount of photosynthetic enzymes per unit leaf area (Li et al., 2011). SPAD value indicates chlorophyll concentration and nitrogen status of the leaves in rice. Although similar LAI and SLW were found among the three lines at heading (**Figures 3A,B**), the SPAD value in R201 was higher than that in R91 and R156, especially at the middle and late grain filling stages (**Figures 3C,D**). Castro et al. (2011) reported that SPAD value is indirectly related with several photosynthetic parameters, such as leaf chlorophyll and Rubisco contents, and apparent photosynthetic rate. Therefore, a higher SPAD value in R201 could imply possible delay in leaf senescence or higher photosynthetic capacity, compared to R91 and R156. Actually, we measured the flag leaf photosynthetic rate (*P*n) of the three lines during grain filling at 2016, and R201 had the highest *P*n, followed by R156 and R91 (19.6 μmol m^-2^ s^-1^ in R201, 18.3 in R156, and 8.6 in R91). Compared to R91 and R156, R201 had the same or higher LAI, SLW, SPAD (**Figures 3A–D**), *P*n, and dry matter accumulation (**Table 1**), and R201 had higher stem and rachis NSC concentration and high NSC ratio of stem to rachis at maturity (**Figures 1B–D**), and higher stem NSC per spikelet (**Figure 6F**), but there was no concomitant advantage accrued in grain filling and yield (**Table 1**). Therefore, the data suggest that it is not possible that functional leaves should be responsible for the low grain filling percentage due to scarcity of photo assimilates.

Additionally, it is noticed that NSC translocation of R201 was negative under LN and very low under HN in comparison to the positive or large values of other two lines (**Figures 2A–C**). Thus, these data imply that stem NSC translocation rather than photosynthetic capacity and supply of carbohydrates in both leaves and stems is the limiting factor for poor grain filling and grain yield formation in R201.

### Candidate Causes for Low NSC Translocation in R201

During grain filling period, many factors, such as enzyme activities driven NSC remobilization in stems (Yang et al., 2001a), phloem loading (Wang et al., 2015), vascular bundle characters (Terao et al., 2010), phloem unloading (Patrick, 1997), and sink activity (Mohapatra et al., 2011), affect translocation and partitioning of stem NSC. In our study, remobilization capability of NSC in stems was reflected by the enzyme activities that are related to the conversion of starch to sucrose in stems. Four key enzymes (α-amylase and β-amylase for starch degradation, SPS and SSs for sucrose synthesis) are involved in starch to sucrose conversion for NSC remobilization in stems after heading. In the present study, the three lines had similar activities of α-amylase, β-amylase, SPS, and SSs at 7 days after heading (**Figures 4A–D**). Additionally, the time course analysis of the four enzyme activities of R156 and R201were performed in another field experiment in 2016, and the data showed that the activities of the four enzymes were not significantly different between the two lines during the entire grain filling stage (Supplementary Figures S1A–D). These results suggest that the three lines have the similar rates of starch degradation and sucrose synthesis, as well as similar remobilization capability of sugars in stems. Therefore, the starch to sucrose conversion in stems may not be the causes for low NSC translocation in R201. Our study did not investigate the loading in stems, however, Yang et al. (2001a) observed that the remobilization capability reflected by the enzyme activities for sucrose synthesis in stems was significantly correlated with rate and amount of NSC translocation. The sequential phloem loading of carbohydrates into vascular bundles for long distance transport should be investigated.

Very recently the close relationships of vascular bundle related traits with yield formation have been documented in rice with identification of *dep1* indica allele in recombinant inbred lines derived from japonica/indica background, the allele exhibited superiority in vascular bundle related traits and yield components (Xu et al., 2015). The linkage between vascular bundle characters and yield formation have been observed, enhanced formation of vascular bundle systems promoted carbohydrate translocation to panicle (Terao et al., 2010). Compared to R91 and R156, R201 had advantages of number and total cross sectional area of LVB (**Figures 5A,E**), and number and total cross sectional area of LVB per spikelet (**Figures 6A,B**) and similarity of the features in SVB (**Figures 5B,F, 6C,D**). Those results indicate that vascular bundles are not the limiting factor for assimilate transport for grain filling and grain yield formation in R201.

Phloem unloading plays pivotal roles in photo assimilates transport and partitioning into sink organs (Patrick, 1997). During grain filling of rice, a developing grain receives assimilates as soluble sugars, mostly in the form of sucrose from the sieve tube terminus of phloem via apoplastic and symplastic pathways (Kuanar et al., 2010; Patrick et al., 2013), following by transformation of sucrose into starch. Considering the advantages of number and total cross sectional area per spikelet of LVB and similarity of the features in SVB, and higher stem and rachis NSC concentration and higher ratio of stem NSC to rachis NSC at maturity (**Figures 1B–D**), and lower NSC translocation in R201, compared to R91 and R156 (**Figures 2A–C**), the supply and transport capacity of stem NSC should not be the limiting factor for poor grain filling. Higher rachis NSC concentration in R201 implies that NSC transported to rachis resided in rachis and cannot be unloaded into the developing grains, and the case may feedback inhibit the NSC transport in stems, resulting in the high NSC accumulation in stems (Mohapatra et al., 2011). Additionally, our preliminary data suggest that R201 has less plasmodesma density in sieve elements-companion cells, compared to R91 and R156 (data not presented). Therefore, a bottleneck in phloem unloading of rachis NSC at sink might have contributed to low NSC translocation, poor grain filling and yield formation in R201.

On the other hand, it might be possible that the unloaded NSC in the form of sucrose could not be used for grain starch on account of poor sink activity of the developing spikelets (Mohapatra et al., 2011). Sink activity (activities of the enzymes related to sucrose to starch conversion in grains) was significantly correlated with grain filling rate, grain weight and grain filling percentage in rice (Yang et al., 2003; Yang and Zhang, 2010b; Zhang et al., 2012). In the study, we investigated the related enzyme activities in developing grains at 7 days after heading. Many previous papers reported that the stem NSC decreased and grain weight increased after anthesis, and time course analysis of grain filling and enzyme activity also showed that grain filling rate and enzymes activities of apical grains on a panicle increased first and then decreased during grain filling period, and the peak value was often observed during the duration of 5–10 days after heading (Yang et al., 2006; Zhu et al., 2011), and the grains had the highest activities of AI (Zhang et al., 2012), sucrose synthase and AGPase (Fu et al., 2011; Li et al., 2012) at the same time. Therefore, the differences in the enzyme activities and NSC concentration among three lines may be reflected to some degree using the observations based on samples at 7 days after heading. Our investigation showed that the three lines had similar activities of the enzymes related to sucrose decomposition (AI, NI, and SSc) (**Figures 7A–C**) and starch synthesis (AGPase) (**Figure 7D**) in developing grains. On the other hand, the time course analysis also showed that the activities of the four enzymes were not significantly different between R156 and R201 during the grain filling stage in another field experiment in 2016, respectively (Supplementary Figures S2A–D). Taken together, the results indicate that three lines have similar sink activity. Therefore, capacity for transforming sucrose into starch should be not limiting factor for low NSC translocation and low grain filling percentage in R201.

As discussed above, our data strongly suggests phloem unloading as candidate cause for low transport of assimilates (including stem NSC translocation) and sequentially low grain filling percentage. The rate of phloem unloading serves as a key determinant of crop yield and plant productivity (Patrick, 1997), and an adaptive shift of phloem unloading pathway during fruit development is beneficial for rapid increase in both berry volume and soluble sugar accumulation (Zhang et al., 2006). However, additional physiological and molecular analyses are required to prove that.

### Effects of Nitrogen on NSC Translocation

In our study, nitrogen application was done in HN to ensure maintenance of high biomass and NSC reserves at heading at the appropriate level for expression of the inherent high yield potential. But the application, although improved yield, failed to impart a corresponding rise in stem and rachis NSC concentration at heading and maturity (**Figures 1A–C**). This observation is consistent with Hirano et al. (2005) and Pan et al. (2011) that fewer carbohydrates were accumulated in stems at heading under high nitrogen. Recently, Deng et al. (2016) partly differed and reported that nitrogen application to rice improves NSC at heading and remobilizes its translocation to grains at later stages of grain development for increase of yield. It is known that rice plant requires carbohydrates to constitute the plant structure, and also needs additional carbon for transformation of the extra inorganic nitrogen absorbed from the growth medium (Gebbing et al., 1999). These biochemical activities may lead to decreases in stem NSC at heading under high nitrogen condition.

Generally, there was no significant difference of NSC translocation between LN and HN in R91 and R156 (**Figures 2A–C**). In the reports of Gebbing and Schnyder (1999) and Pan et al. (2011), LN enhanced the NSC remobilization and its contribution to grain filling and yield formation. Contrarily, nitrogen application significantly increased NSC translocation in R201 (**Figures 2A–C**). Experimental conditions were usually different in different studies, for example, variety, water supply, and nitrogen rate, which may have great influence on plant growth, leaf function, carbohydrates re-location, and yield formation. Water stress treatment increased stem NSC translocation by 36.7% under high nitrogen condition, which was higher than 27.1% under low nitrogen condition in rice variety of Wuyujing 3, when compared to well water treatment. However, in variety of Yangdao 6, NSC translocation increased by 19.6% under high nitrogen condition, which was lower than 23.8% under low nitrogen condition (Yang et al., 2001b). Moreover, in maize, He et al. (2001) reported that an appropriate N level was beneficial for NSC translocation from vegetative parts to grains, and therefore, achieved the highest yield, N omission and excessive N was not conducive to NSC accumulation and translocation and could not get the highest yield. Those previous observations suggest that variety, nitrogen applicant rate and its interaction with water supply influence stem NSC translocation. Optimal field management practices such as N application rate before flowering should receive more attention to improve NSC translocation and yield formation.

## Conclusion

The results in present study showed that R201 had lower grain yield than that of R91 and R156, which was attributed to low grain filling percentage of R201, and spikelet number per panicle and spikelet fertility had no effect on grain filling percentage (**Table 1**). The results indicated that photosynthetic capacity and supply of carbohydrates was not the limiting factor for grain filling in R201 (**Figures 3A–D**). On the other hand, tightly positive association between stem NSC translocation and grain filling percentage was observed in the three lines (**Figures 2A–C** and **Table 1**), which suggested that low NSC translocation from stems to grains might result in low grain filling percentage in R201. R201 had higher values of LVB related traits (**Figures 5A,E, 6A,B**), higher the stem and rachis residues of NSC (**Figures 1B–D**), similarity of SVB related traits (**Figures 5B,F, 6C,D**), and similarity of the remobilization capability of assimilation in stems (**Figures 4A–D**) and the same sink activities (**Figures 7A–D**), compared to R91 and R156. Therefore, low NSC translocation, poor grain filling and yield formation in R201 might be attributed to an obstacle in phloem unloading of NSC from transport system to grains. The mechanisms underlying of the low phloem unloading in R201 merit further researches.

## Author Contributions

GL and KC conceived the research, designed experiments, analyzed the data and wrote the manuscript. JP selected the three lines of rice. GL carried out field experiments. MY assisted in both sampling and measurements in the field. QH and WW assisted in physiological determination in the laboratory. PM revised the manuscript. SP, JH, and LN gave valuable suggestions during the whole field and laboratory experiments.

## Conflict of Interest Statement

The authors declare that the research was conducted in the absence of any commercial or financial relationships that could be construed as a potential conflict of interest.

## Supplementary Material

FIGURE S1 Activities of α-amylase **(A)**, β-amylase **(B)**, sucrose phosphate synthase **(C)** and sucrose synthase in the synthetic direction **(D)** in stems of rice R156 and R201 under low (LN) and high nitrogen (HN) conditions during grain filling period. Vertical bars indicate LSD_0.05_ for comparing the means between the two lines at an identical stage. Heading date was defined as the day that 50% panicles had exserted; DAH, day after heading. Data are shown as mean ± standard error.

FIGURE S2 Activities of acid invertase **(A)**, neutral invertase **(B)**, sucrose synthase in the cleavage direction **(C)** and AGPase **(D)** in developing grains of rice R156 and R201 under low (LN) and high nitrogen (HN) conditions. Vertical bars indicate LSD_0.05_ for comparing the means between the two lines at an identical stage. Heading date was defined as the day that 50% panicles had exserted; DAH, day after heading. The date of 33 DAH was the date for harvest. Data are shown as mean ± standard error.

## Abbreviations

AC: apparent contribution of transferred NSC to grain yield
AGPase: adenosine diphosphate-glucose pyrophosphorylase
AI: acid invertase
AR: apparent ratio of transferred NSC from stems to grains
ATM: apparent transferred mass of NSC from stems to grains
HN: high nitrogen application
LAI: leaf area index
LN: low nitrogen application
LVB: large vascular bundle
NI: neutral invertase
NSC: non-structural carbohydrates
QTLs: quantitative trait loci
SLW: specific leaf weight
SPS: sucrose phosphate synthase
SSc: sucrose synthase in the cleavage direction
SSs: sucrose synthase in the synthetic direction
SVB: small vascular bundle.
