# Supplementary figures and images for: Limitation of Unloading in the Developing Grains Is a Possible Cause Responsible for Low Stem Non-structural Carbohydrate Translocation and Poor Grain Yield Formation in Rice through Verification of Recombinant Inbred Lines
Source: Front Plant Sci. 2017 Aug 8;8:1369. doi: 10.3389/fpls.2017.01369 (PMC5550689; doi:10.3389/fpls.2017.01369)

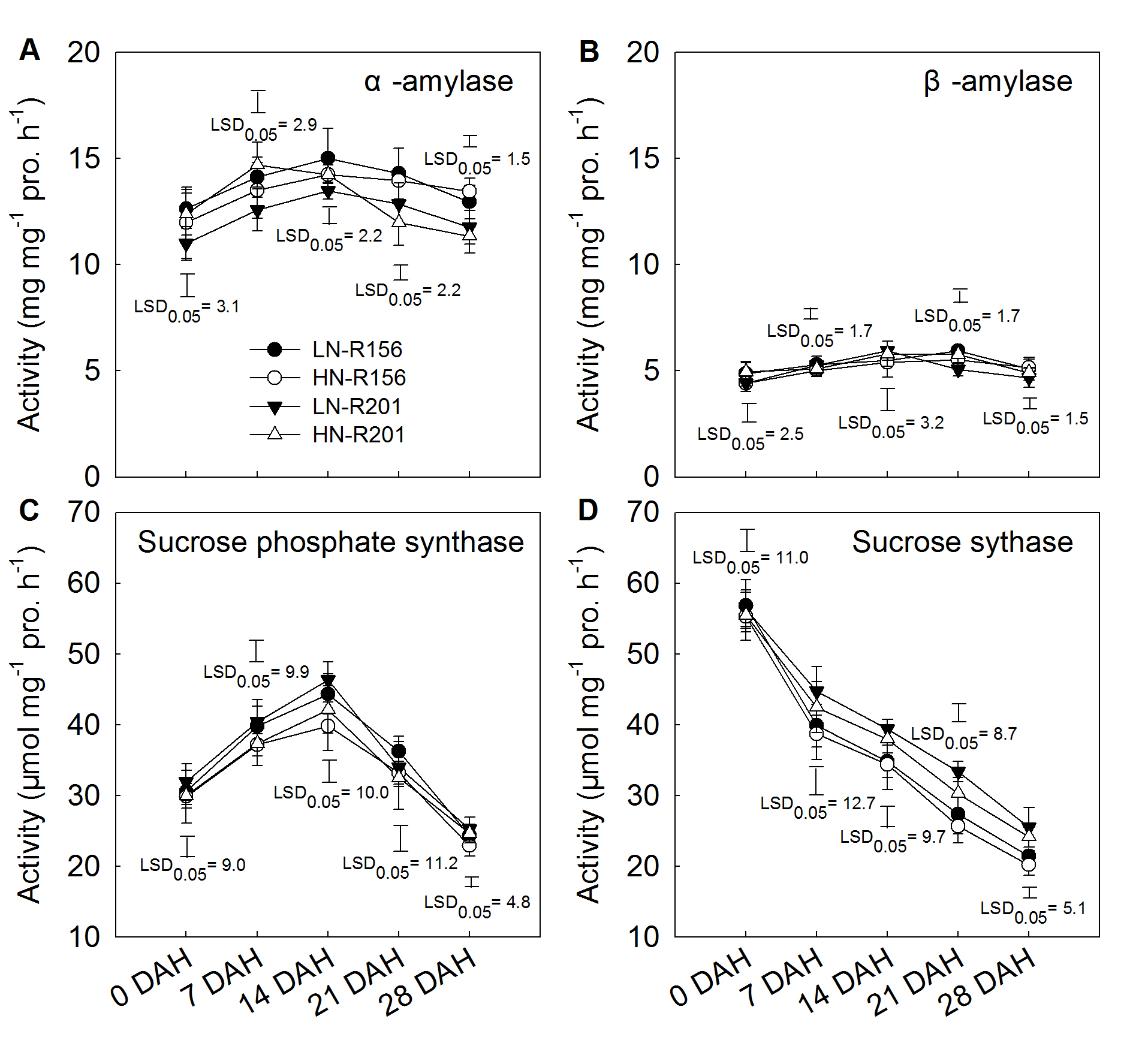

Supplement: FIGURE S1 — Activities of α-amylase (A), β-amylase (B), sucrose phosphate synthase (C) and sucrose synthase in the synthetic direction (D) in stems of rice R156 and R201 under low (LN) and high nitrogen (HN) conditions during grain filling period. Vertical bars indicate LSD0.05 for comparing the means between the two lines at an identical stage. Heading date was defined as the day that 50% panicles had exserted; DAH, day after heading. Data are shown as mean ± standard error. [file Image_1.JPEG]

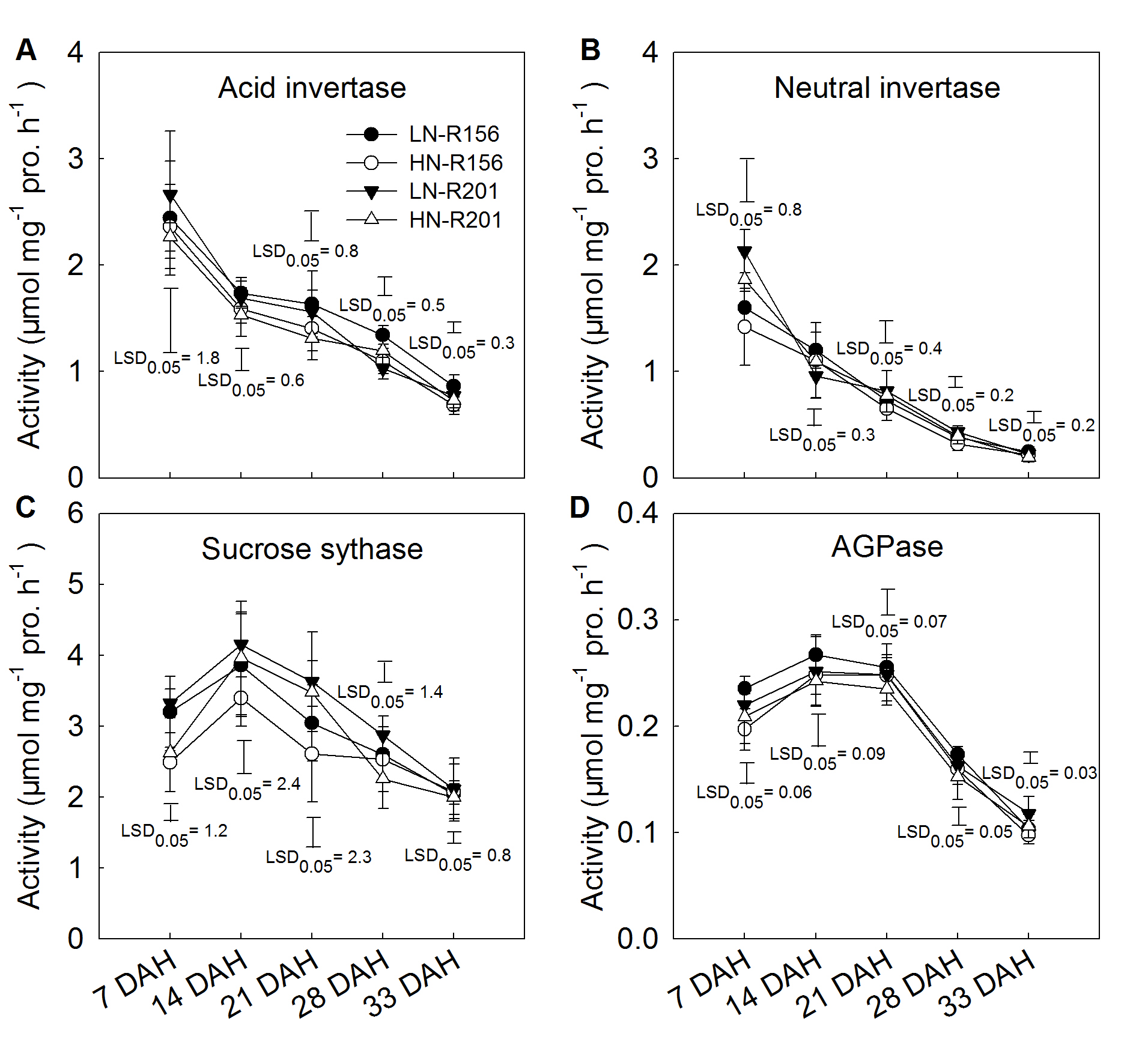

Supplement: FIGURE S2 — Activities of acid invertase (A), neutral invertase (B), sucrose synthase in the cleavage direction (C) and AGPase (D) in developing grains of rice R156 and R201 under low (LN) and high nitrogen (HN) conditions. Vertical bars indicate LSD0.05 for comparing the means between the two lines at an identical stage. Heading date was defined as the day that 50% panicles had exserted; DAH, day after heading. The date of 33 DAH was the date for harvest. Data are shown as mean ± standard error. [file Image_2.JPEG]
