# Supplementary material for: Limitation of Unloading in the Developing Grains Is a Possible Cause Responsible for Low Stem Non-structural Carbohydrate Translocation and Poor Grain Yield Formation in Rice through Verification of Recombinant Inbred Lines
Source: Front Plant Sci. 2017 Aug 8;8:1369. doi: 10.3389/fpls.2017.01369 (PMC5550689; doi:10.3389/fpls.2017.01369)
Supplement: Supplementary file 3 [file Table_1.docx]

**Supplementary Table S1**

**Absorbance value of solution with substrate involved in enzyme activity determination and with water instead of substrate**

| Substrate | OD-Substrate | OD-H_2_O |
| --- | --- | --- |
| UDP-glucose for SPS and SSs | 0.05 ± 0.00 ^a^ | 0.05 ± 0.00 ^a^ |
| Sucrose for AI, NI and SSc | 0.11 ± 0.00 ^a^ | 0.11 ± 0.00 ^a^ |
| ADP-glucose for AGPase | 0.19 ± 0.00 ^a^ | 0.19 ± 0.00 ^a^ |

OD-Substrate indicates the absorbance value of substrate solution without enzyme extraction after coloration according to the corresponding method described in the section of “Materials and Methods”, respectively. All chemicals were purchased form Sigma Chemical Company (St. Louis, USA). Similarly, OD-H_2_O indicates the absorbance value of distilled water without enzyme extraction. Different letters following mean ± standard derivation (n=3) indicate significant difference between OD-Substrate and OD-H_2_O at p<0.05.

According to **Supplementary Table S1,** all substrates were 100% free of the expected products. Therefore, using enzyme extraction without substrate as “controls” is appropriate.

**Supplementary Table S2**

**Comparison of enzyme activities measured using different controls** **in R156 under low nitrogen condition**

| Enzyme | Activity ( μmol mg^-1^ pro. h^-1^) | |
| --- | --- | --- |
|  | Method 1(CK - H_2_O) | Method 2(CK - inactive enzyme) |
| Stem SPS | 40.7 ± 5.1 ^a^ | 38.2 ± 3.9 ^a^ |
| Stem SSs | 47.5 ± 2.1 ^a^ | 44.9 ± 3.3 ^a^ |
| Grain AI | 2.4 ± 0.3 ^a^ | 2.5 ± 0.2 ^a^ |
| Grain NI | 1.5 ± 0.2 ^a^ | 1.6 ± 0.2 ^a^ |
| Grain SSc | 3.2 ± 0.4 ^a^ | 3.3 ± 0.5 ^a^ |
| Grain AGPase | 0.2 ± 0.0 ^a^ | 0.2 ± 0.0 ^a^ |

According to the methods described in the section of “Materials and Methods”, R156 grown under low nitrogen condition were used to extract the enzymes at 7 d after heading in another field experiment in 2016, respectively. Determination of enzyme activity was carried out according to the procedure described in the section of “Materials and Methods”.

Different letters following mean ± standard derivation (n=3) indicate significant difference between Method 1 and Method 2 at p<0.05. SPS, sucrose phosphate synthase; SSs, sucrose synthase in the synthetic direction; AI, acid invertase; NI, neutral invertase; SSc, sucrose synthase in the cleavage direction; AGPase, adenosine diphosphate-glucose pyrophosphorylase.

Method 1(CK-H_2_O) indicates that the control was conducted using H_2_O instead of substrate, which is t used in our study, and method 2(CK-inactivated enzyme) indicates that the control was conducted using substrate plus enzyme inactivated by placing tube containing enzyme extract into boiling water for 1 min.

According to **Supplementary Table S2,** the data indicate that enzyme activities were not significantly different between Method 1 and Method 2. Therefore, using enzyme extraction without substrate as “controls” is appropriate, and do not result in overestimation or [underestimation](http://www.so.com/link?url=http%3A%2F%2Fdict.youdao.com%2Fsearch%3Fq%3Dunderestimation%26keyfrom%3Dhao360&q=under+estimation&ts=1500549174&t=4649fb2d74bfc719219ec3843dc1df8) for enzyme activity.
